# Assessing implementation fidelity of a community-based infant and young child feeding intervention in Ethiopia identifies delivery challenges that limit reach to communities: a mixed-method process evaluation study

**DOI:** 10.1186/s12889-015-1650-4

**Published:** 2015-04-01

**Authors:** Sunny S Kim, Disha Ali, Andrew Kennedy, Roman Tesfaye, Amare W Tadesse, Teweldebrhan H Abrha, Rahul Rawat, Purnima Menon

**Affiliations:** International Food Policy Research Institute, 2033 K Street, NW Washington, DC 20006 USA; International Food Policy Research Institute, IFPRI-ESARO-ILRI Campus, Addis Ababa, Ethiopia; Addis Continental Institute of Public Health, Road 8, Zone 8, Yeka Subcity, Addis Ababa, Ethiopia; FHI 360, House No. 193, Kebele 02, Kirkos Subcity, Addis Ababa, Ethiopia; International Food Policy Research Institute, Titre 3396, Lot #2, BP 24063 Dakar Almadies, Senegal; International Food Policy Research Institute, NASC Complex, CG Block, Dev Prakash Shastri Road, Pusa, New Delhi, 110012 India

**Keywords:** Implementation fidelity, Process evaluation, Program impact pathway, Infant and young child feeding, Ethiopia

## Abstract

**Background:**

Program effectiveness is influenced by the degree and quality of implementation, thus requiring careful examination of delivery processes and how the program is or is not being implemented as intended. Implementation fidelity is defined by *adherence* to intervention design, *exposure* or dose, *quality* of delivery, and participant *responsiveness.* As part of the process evaluation (PE) of Alive & Thrive in Ethiopia, a large-scale initiative to improve infant and young child feeding (IYCF), we assessed these four fidelity elements along three components of its community-based intervention: training of frontline workers (FLWs), delivery of program tools and messages, and supportive supervision.

**Methods:**

Data from a qualitative study among three levels of FLWs (n = 54), i.e. supervisors, health extension workers (HEWs), and community volunteers, and among mothers with children under two years of age (n = 60); and cross-sectional PE surveys with FLWs (n = 504) and mothers (n = 750) in two regions (Tigray and SNNPR) were analyzed to examine program fidelity.

**Results:**

There was strong adherence to the intended cascading design (i.e. transfer of knowledge and information from higher to lower FLW levels) and high exposure to training (95% HEWs and 94% volunteers in Tigray, 68% and 81% respectively in SNNPR). Training quality, assessed by IYCF knowledge and self-reported capacity, was high and increased since baseline. Job aids were used regularly by most supervisors and HEWs, but only 54% of volunteers in Tigray and 39% in SNNPR received them. Quality of program message delivery was lower among volunteers, and aided recall of key messages among mothers was also low. Although FLW supervision exposure was high, content and frequency were irregular.

**Conclusions:**

There is evidence of strong fidelity in training and delivery of program tools and messages at higher FLW levels, but gaps in the reach of these to community volunteers and mothers and variability between regions could limit the potential for impact. Strengthening the linkages between HEWs and volunteers further can help to reach the target households and deliver IYCF results at scale.

## Background

Child malnutrition persists in Ethiopia, with high rates of childhood stunting (44%) and underweight (29%) [1], characterized by poor infant and young child feeding (IYCF) practices among other underlying factors. In addition, there are challenges in health service delivery through the existing health system, demonstrated by low coverage of essential services such as full childhood vaccination (24%) and any antenatal care during pregnancy (43%) [1].

To scale up primary health care services, the Federal Ministry of Health’s flagship Health Extension Program has deployed government-salaried female health extension workers (HEWs) to health posts in rural communities since 2003 [2-6]. HEWs deliver 17 service packages, including child survival interventions, maternal and neonatal care, nutrition interventions, and hygiene and environmental sanitation measures. Networks of community volunteers were also established to support the HEWs and facilitate activities of health and nutrition promotion and community mobilization. Studies in various regions of the country have shown successes attributed to the Health Extension Program, such as increased vaccination coverage [7]; improved women’s utilization of family planning, antenatal care, and HIV testing services [8]; and improved maternal and newborn health care practices [9]. However, there was little effect on institutional or skilled delivery, use of postnatal services [7,10], some newborn health care practices [9], and health outcomes such as the incidence and duration of childhood diarrhea and cough [7]. These variable results have been ascribed to implementation challenges, particularly poor quality and low availability of some services due to weak technical capacity, inadequate infrastructure and management capacity, and poor monitoring and supervision [10,11]. Thus, impacts may not be achieved despite being interventions of proven efficacy due to programmatic constraints, highlighting the need to examine delivery processes and to understand how the program is or is not being implemented as intended.

Program effectiveness is influenced by the degree and quality of implementation [12-15]. A major reason for program failure even among sound theory-based interventions is the failure to implement with fidelity [12]. A meta-analysis of studies from various fields showed that programs with better implementation had mean effect sizes two to three times larger than those with poor implementation [16]. However, conventional impact evaluations do not focus on the process of program delivery nor sufficiently illuminate the reasons behind the success or failure of interventions. There are few empirical studies of implementation fidelity of nutrition interventions, particularly in developing countries, and a handful of these are process evaluations on fidelity aspects of interventions delivered at health centers. For instance, a study in Peru examined the fidelity of nutrition education provided by health staff, which showed that increased caregiver exposure, despite relatively low quality and adherence to protocol, led to improvements in specific message recall and child feeding behaviors [17]. The purpose of our study is to assess implementation fidelity along the continuum of the delivery process of a community-based IYCF intervention in Ethiopia, to inform program progress toward impact.

### Implementation fidelity conceptual framework

Implementation fidelity, or program integrity, is the degree to which programs are implemented as intended [13,15,18,19]. We adapt four common elements of implementation fidelity: *adherence* to intervention design; *dosage and exposure*; *quality of delivery*; and *participant responsiveness* [15]. Adherence is defined as whether “a program, service or intervention is being delivered as it was designed” [13]. Dosage (dose delivered) and exposure (dose received) refers to “whether the frequency and duration of the intervention is as full as prescribed” [12,13] and includes coverage, i.e. how many of the targeted beneficiaries actually receive benefits or participate. Quality of delivery refers to how well the staff delivers a program [13]. Participant responsiveness “measures how far participants respond to or are engaged by an intervention” [15]. Furthermore, rather than considering each of these elements as either alternative measures or part of a composite measure, Carroll et al. conceptualized adherence (including content, frequency, duration, and coverage) as the central measurement of fidelity, and delivery quality, participant responsiveness, and other factors as moderators [15]. We apply this categorization in our analysis and interpretation of results.

### Description of A&T Ethiopia’s community-based IYCF intervention

Alive & Thrive (A&T) Ethiopia is a multi-year initiative that started in 2009 and aimed at reducing undernutrition caused by suboptimal breastfeeding and complementary feeding practices. The program delivers age-appropriate child feeding messages and counseling to mothers and caregivers of children less than two years of age at the community level primarily through the Health Extension Program, utilizing the large network of HEWs and community volunteers. Coverage of A&T community-based interventions is intended to be achieved at scale through different program platforms in the four most populous regions, i.e. Amhara; Oromia; Southern Nations, Nationalities and Peoples Region (SNNPR); and Tigray. These platforms include a direct partnership with the USAID-funded Integrated Family Health Program (IFHP), which covers more than 80% of A&T program areas, as well as partnerships with other local organizations such as faith-based organizations and women’s associations to provide services to the target population and mobilize communities. Also, there are region-specific partners such as the large nongovernmental organization in Tigray called the Relief Society of Tigray (REST). At the community level, we refer generically to the cadre of frontline workers (FLWs) as community volunteers, previously called volunteer community health promoters, which were scaled up and restructured by government policy in 2012 to the Women’s Development Armies^a^ (WDAs) or Health Development Armies (HDAs). In addition to the community-based intervention, a large scale mass-media campaign in various local languages was launched in the four regions to promote IYCF messages through radio, TV, and mobile vans to show video clips.

**Figure 1:**
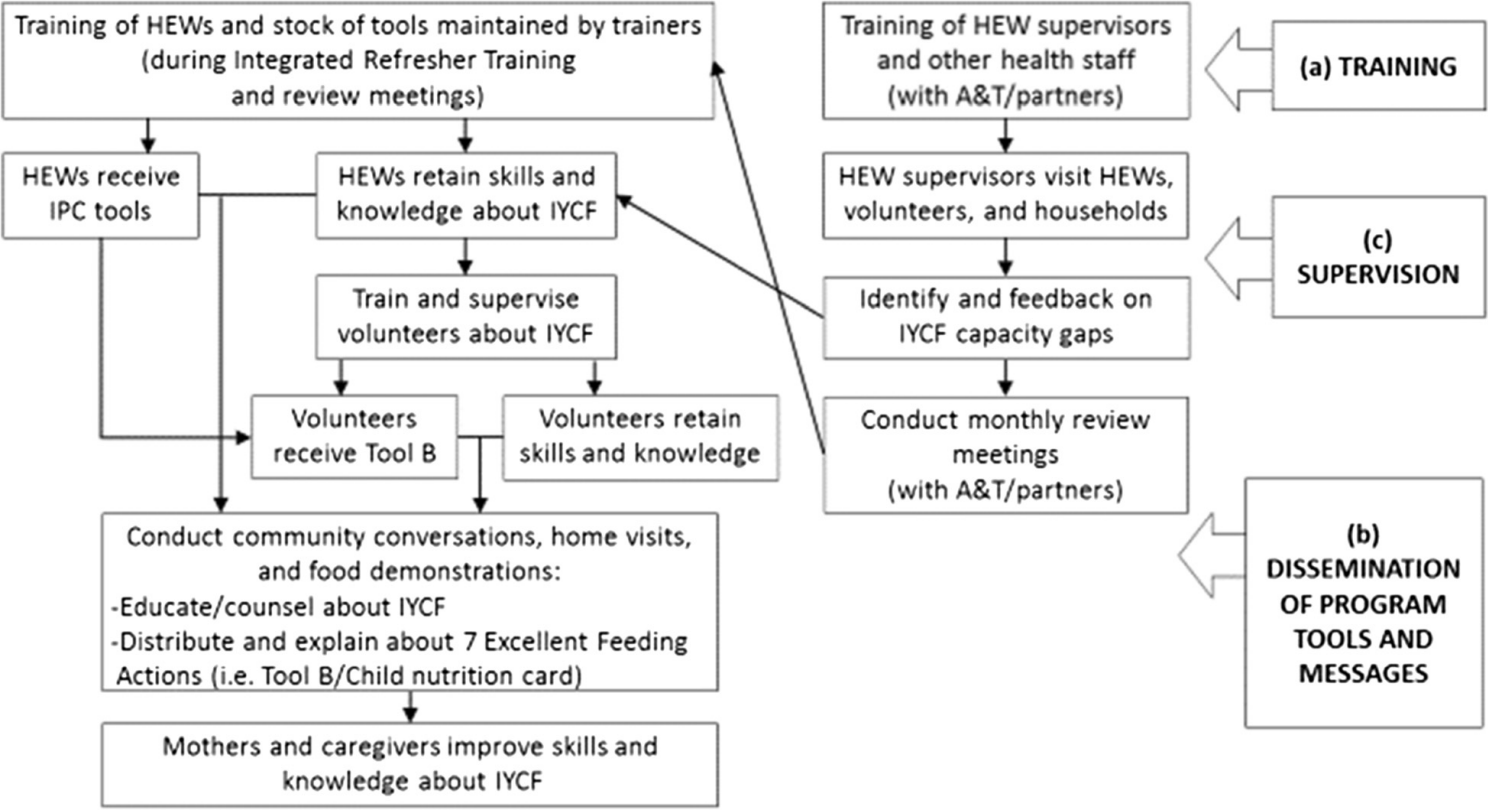
**Program impact pathways for A&T Ethiopia’s community-based intervention.** NOTE: A&T’s interpersonal communication (IPC) tools on IYCF (i.e. Tool B/child nutrition card) contain the key program messages about the “7 Excellent Feeding Actions”.

The central approach of the community-based intervention is the cascading schema, involving the transfer of information, knowledge and skills, as well as program tools from FLWs at higher to lower levels (i.e. from HEW supervisors and HEWs to volunteers). FLW capacity building is a major focus of the program, and health workers should receive enhanced training (at least once) and refresher training on nutrition with a particular focus on IYCF, as per the manual and tools developed by A&T. Then FLWs disseminate the program tools and provide education and interpersonal counseling to mothers and caregivers in the community, during health post visits, home visits, or community gatherings. HEWs and volunteers receive monthly supportive supervision and feedback in order to identify program gaps and reinforce capacities. Fulfilment of the program delivery as above is intended to lead to optimal IYCF practices and final impact of improved child nutritional status. To highlight the scope of this study, Figure 1 presents the delivery side of the A&T program impact pathways, outlining the three programmatic components of (a) training, (b) dissemination of program tools and messages, and (c) supervision of the community-based intervention.

In relation to the timeline of implementation, initial training of HEWs in some program intervention areas started in August 2010. However, the Federal Ministry of Health instituted the policy of government-managed Integrated Refresher Training (IRT) in June 2011, which stipulated that HEWs were only to receive training through IRTs. As a result, A&T incorporated IYCF information and materials into the maternal and child health module^b^ of the IRT, and training of trainers and all HEWs on this module was completed by September 2012. In parallel to IRT rollout, A&T/partners provided (1) primary healthcare staff training for supervisors and health workers (to provide on-the-job training to HEWs and other FLWs); (2) training and provision of supportive supervision that includes IYCF; and (3) review meetings to provide additional training or refreshers based on knowledge gaps identified during supervision. Also in mid-2011, a policy for restructuring the volunteer community health promoters into the WDA/HDA was put in place, and the new cadre of volunteers was established in 2012.

## Methods

### Study context

This study is part of a larger process evaluation (PE) of A&T’s IYCF interventions conducted in two regions (SNNPR and Tigray) of Ethiopia [20], within the framework of an impact evaluation with adequacy design that captures changes over time, in the absence of a control group [21,22]. First, a qualitative PE study among FLWs and mothers to examine perspectives on program implementation was conducted between May and July 2012. Then between April and June 2013, quantitative PE surveys were conducted with FLWs and households to assess program reach, service provision and utilization.

### Sampling and data collection

For the qualitative study, six *woredas* (districts), three per region, were purposively selected for differences in A&T platforms or partners and operational duration, in order to capture variations in program delivery processes. In each woreda, two *kebeles* (villages) were selected randomly. Primary data collection involved in-depth semi-structured interviews (n = 54) with HEW supervisors (one per woreda), HEWs (one per kebele), and community volunteers (three per kebele), as well as brief interviews with beneficiaries (n = 60) to triangulate responses of FLWs and verify program exposure. Interviews were audio recorded and transcribed by the interviewers, then translated into English for data analysis.

PE surveys for FLWs and households were conducted in the 75 enumeration areas (26 in Tigray and 49 in SNNPR) in 55 woredas that are part of the impact evaluation. Household surveys (n = 750) were conducted among mothers, using a two-stage cluster sampling method (enumeration area selected based on probability proportion to size sampling in the first stage, and households with children under two years of age as the second stage of sampling). In each enumeration area/kebele, there is usually one health post, which consists of two HEWs. Both HEWs, their supervisor, and approximately four volunteers from a list of community health volunteers identified by HEWs in each enumeration area were selected to participate in the FLW survey (n = 504). A summary of the topics covered and the sample type and size for each data collection method is provided in Table 1. Separate structured questionnaires were applied for different FLW types, and questions covered topics such as exposure to nutrition and IYCF-related training, utilization of interpersonal communication (IPC) tools promoted by the program, exposure to different program strategies, knowledge about IYCF, work motivation and self-efficacy. The household questionnaire focused on exposure to program strategies and IPC tools and on IYCF knowledge and practice.

**Table 1:** **Data collection methods and sample**

**Method (Year)**	**Data collection topics**	**Sample type and size**
Semi-structured interview with FLW (2012)	Mapping of program pathways and factors influencing service delivery; perceptions of and exposure to training, program tools, and supervision; and perceptions of workload, job satisfaction and motivation	54 FLWs (6 supervisors, 12 HEWs, and 36 volunteers) in 6 woredas (3 per region)
Semi-structured interview with mother (2012)	Perceptions of and exposure to FLW contacts, program tools and activities	60 mothers with children aged 0–23.9 months in 6 woredas (3 per region)
FLW survey (2013)	Exposure to training and supervision; utilization of interpersonal communication tools; time commitments to IYCF counseling; IYCF knowledge; and work motivation and self-efficacy	504 FLWs (75 supervisors, 150 HEWs, and 279 volunteers) in 55 woredas across both regions
Household survey (2013)	Exposure to program activities and interpersonal communication tools; and IYCF knowledge and practice	750 mothers with children aged 0–23.9 months in 55 woredas across both regions

Results from the 2013 PE survey were compared with the 2010 baseline survey data, where similar measures are available such as FLW training exposure and IYCF knowledge. Although similar sampling methods and instruments were used, there were differences in sampling frames and sizes between the two cross-sectional surveys (e.g. baseline included households with children under five years of age, thus more households, and a smaller sample of FLWs).

### Data analysis

Quantitative data were analyzed using Stata 12 [23]. Descriptive statistics were used to describe the sample characteristics of FLWs and mothers, as well as to present percentage distributions of indicators of fidelity elements by respondent type and region. For indicators of IYCF knowledge, un-weighted summary scores were constructed from several key items that constitute knowledge about breastfeeding (BF) or complementary feeding (CF), measured at both baseline and PE. A simple additive score for the knowledge type (BF or CF) was created, summing each correct response to key questions. The percentage of each correct item/response and the overall mean scores for knowledge types are presented. For comparisons between the PE survey and the baseline survey data, trends and patterns in the results are described. No statistical tests were applied. Content analysis of the interview transcripts was conducted using the qualitative data analysis software NVivo 10 [24]. Transcripts were systematically coded and analyzed by a team of standardized coders, using a detailed a priori thematic code list based on the research protocol and instruments which were refined and supplemented with emergent themes. Outputs of code queries were interpreted and summarized for findings related to fidelity elements.

Results are presented by the three programmatic components of training, dissemination of program tools and messages, and supervision. For each component, we show results of the fidelity elements of adherence, exposure, quality and participant responsiveness. Given that various measures were used to assess each element of implementation fidelity, not all of the data tables and figures are included in this paper, although the main results are discussed.

The study protocol was approved by the Institutional Review Boards of the Ministry of Science and Technology in Ethiopia and of the International Food Policy Research Institute. Written informed consent was obtained from all study participants.

## Results

### Sample characteristics

Table 2 presents the characteristics of FLWs from the quantitative survey and qualitative study by type and region. Characteristics of supervisors (n = 25 in Tigray and n = 46 in SNNPR), HEWs (n = 40 and n = 93), and volunteers (n = 104 and n = 196) were similar between regions. HEWs and volunteers generally worked longer in their current positions than the supervisors. A total of 750 mothers with children under two years of age (n = 260 in Tigray and n = 490 in SNNPR) were included in the household survey. Most mothers’ characteristics were similar between regions (mean age, education, marital status, and occupation).

**Table 2:** **Characteristics of samples from the 2013 PE survey and 2012 qualitative study**

	**Supervisors**		**HEWs**		**Volunteers**		**Mothers**	
**PE SURVEY SAMPLE, 2013 indicators**	**Tigray (n = 25)**	**SNNPR (n = 46)**	**Tigray (n = 40)**	**SNNPR (n = 93)**	**Tigray (n = 104)**	**SNNPR (n = 196)**	**Tigray (n = 260)**	**SNNPR (n = 490)**
	**Percent/Mean (SD)**	**Percent/Mean (SD)**	**Percent/Mean (SD)**	**Percent/Mean (SD)**	**Percent/Mean (SD)**	**Percent/Mean (SD)**	**Percent/Mean (SD)**	**Percent/Mean (SD)**
Age (years)	33.0 (7.8)	25.0 (2.3)	27.1 (5.7)	25.2 (3.1)	33.8 (9.1)	30.9 (7.6)	27.8 (0.5)	27.4 (0.5)
Number of children:								
0	36.0	63.2	22.5	30.7	5.8	8.3	0.0	0.0
1	28.0	23.7	50.0	42.1	7.7	6.2	38.5	42.2
2	4.0	7.9	20.0	17.1	22.1	8.3	57.1	48.5
3+	32.0	5.3	7.5	10.2	64.4	77.3	4.4	9.3
Highest education level completed:								
Nursing, university	100.0	100.0						
Technical/vocational			60.0	63.4				
High school			37.5	26.9				
Secondary school			2.5	4.3	9.5	35.1	5.8	6.3
Grade 1-8			0.0	5.4	49.4	53.6	35.4	53.0
Barely read or write/illiterate					41.4	11.3	58.8	40.7
Years in current position	3.5 (2.4)	1.6 (1.6)	4.1 (0.6)	4.4 (0.2)	4.1 (3.7)	4.6 (3.6)	N/A	N/A
**QUALITATIVE STUDY SAMPLE, 2012**	**Tigray (n = 3)**	**SNNPR (n = 3)**	**Tigray (n = 6)**	**SNNPR (n = 6)**	**Tigray (n = 18)**	**SNNPR (n = 18)**	**Tigray (n = 30)**	**SNNPR (n = 30)**
Age (years)	31.0 (9.4)	29.3 (4.9)	25.2 (1.2)	25.2 (2.3)	32.9 (9.4)	32.9 (6.5)	25 (4.6)	25 (4.6)
Highest education level completed:								
Nursing, university	66.7	66.7						
Technical/vocational	33.3	33.3	100.0	83.3				
High school			0.0	0.0	0.0	16.7	0.0	3.3
Secondary school			0.0	16.7	11.1	55.5	13.3	23.3
Grade 1-8					83.3	27.8	43.3	43.3
Barely read or write/illiterate					5.6	0.0	43.3	30.0
Years in current position	1.4 (1.4)	1.6 (1.2)	6.0 (0.5)	3.8 (1.6)	4.2 (6.1)	5.1 (3.1)	N/A	N/A

A total of 54 FLWs were interviewed for the qualitative study in Tigray (n = 27) and SNNPR (n = 27), with equal sample sizes across the six different woredas (Table 2). FLW characteristics were similar between the woredas and the regions. HEWs and volunteers included in the qualitative study had slightly higher education levels than the survey sample, but patterns of other characteristics were similar. Ten mothers with children under two years of age were interviewed in each woreda for a total of 60 mothers, equally in Tigray and SNNPR; eight mothers in Tigray and three in SNNPR had children under six months of age. Mothers in the qualitative study were slightly younger and more educated than those in the survey sample.

### Training of frontline workers (Figure 1, component a)

#### Training adherence and exposure

Capacity building of FLWs is the main focus of A&T collaboration with partner organizations (e.g. IFHP and REST). The A&T enhanced training about child nutrition and IYCF is called ENA-BCC-CF (Essential Nutrition Actions-Behavior Change Communication-Complementary Feeding), which enhances the prior training on ENA-BCC that includes BF information. ENA-BCC-CF content was incorporated into the maternal and child health module of IRT, which was rolled out to all HEWs. Additional training or refreshers are also provided by A&T/partners to supervisors and HEWs on a needs basis during review meetings for various health programs, in coordination with the Regional Health Bureaus. Then HEWs develop their plans for training and orienting the community volunteers, in order to transfer their knowledge and expand coverage of IYCF counseling and education in the communities.

Given potential exposure to similar training content from different sources, unique A&T program “tracers” such as the “7 Excellent Feeding Actions” (A&T’s key IYCF messages) and specific content items (e.g. recommendation of dried meats as animal-source foods and food demonstration practices) were used to determine adherence to content and verify types of training received. Most FLWs confirmed having received nutrition training, specifically ENA-BCC-CF training (80.0% and 38.1% of supervisors in Tigray and SNNPR respectively, 95.0% and 67.7% of HEWs, and 93.8% and 81.4% of volunteers) (Table 3). Training exposure was lower in SNNPR than in Tigray for all FLW types. Compared to baseline, there was a substantial increase from exposure to previous ENA-BCC training. However, Community-Based Nutrition (CBN), a program introduced within the Health Extension Program in 2008 with the support of UNICEF and the World Bank, was also identified as a major training source among supervisors and HEWs. Responses were related to at least one training event, since frequency of more than one session of any training was low and varied.

**Table 3:** **Nutrition training received by frontline workers by region and survey year**

**Indicators**	**2010**		**2013**	
	**Percent**		**Percent**	
	**Tigray**	**SNNPR**	**Tigray**	**SNNPR**
**Supervisors**	**(n = 25)**	**(n = 47)**	**(n = 25)**	**(n = 46)**
Received any training on nutrition	92.0	68.1	96.0	63.5
In past one year	N/A	N/A	44.0	43.5
Training on ENA-BCC^1^/ENA-BCC-CF^2^	52.0	28.9	80.0	38.1
Training on Community based nutrition (CBN)	88.0	37.8	64.0	28.6
Source of any nutrition training:				
IFHP/A&T	N/A	N/A	54.2	37.9
REST	N/A	N/A	16.7	0.0
CBN	N/A	N/A	12.5	24.1
Others/NGOs	N/A	N/A	16.7	34.5
	**Tigray**	**SNNPR**	**Tigray**	**SNNPR**
**HEWs**	**(n = 25)**	**(n = 48)**	**(n = 40)**	**(n = 93)**
Received Integrated Refresher Training (IRT)	N/A	N/A	95.0	95.7
Received any training on nutrition	96.0	79.2	95.0	95.7
In the past one year	N/A	N/A	42.1	49.4
Training on ENA-BCC^1^/ENA-BCC-CF^2^	12.0	18.8	95.0	67.7
Training on CBN	100	41.7	92.5	67.7
Sources of training on ENA-BCC-CF:				
IRT	N/A	N/A	13.2	7.9
IFHP/A&T	N/A	N/A	60.5	77.8
REST	N/A	N/A	26.3	0.0
Others (women’s association, local NGOs)	N/A	N/A	21.0	19.1
	**Tigray**	**SNNPR**	**Tigray**	**SNNPR**
**Volunteers**	**(n = 25)**	**(n = 48)**	**(n = 104)**	**(n = 196)**
Received any training on nutrition	96.2	89.8	92.3	85.1
Training on ENA-BCC^1^/ENA-BCC-CF^2^	3.9	6.1	93.8	81.4
Training on CBN	80.8	38.8	69.8	65.9
Source of training on ENA-BCC-CF:				
HEW supervisors	N/A	N/A	54.6	22.6
HEWs	N/A	N/A	70.5	69.1
Others/NGOs	N/A	N/A	16.2	20.4

^1^Training on ENA-BCC (essential nutrition actions, which include breastfeeding messages but not specifically on overall IYCF) was being conducted by IFHP and other partners prior to 2010 baseline survey.

^2^Through A&T, IFHP and other partners introduced ENA-BCC-CF, a training curriculum with special emphasis on IYCF, particularly complementary feeding (CF).

As intended by program design, training on nutrition was received by the community volunteers primarily from HEWs (70.5% in Tigray and 69.1% in SNNPR). The importance of reaching the volunteers to train and work with them was reiterated by supervisors and HEWs: “*I took the initiative to give training because they [volunteers] are supportive… because I and all the HEWs cannot reach all the households, educating and assigning to each WDA is a better approach. What we usually transmit then spreads to the grassroots level very quickly*.”

#### Training quality and responsiveness

Training quality was measured distally in terms of IYCF knowledge (Table 4) and self-efficacy in providing IYCF education to beneficiaries (data not shown). BF and CF knowledge increased among supervisors and HEWs in both regions, although BF and specific CF knowledge already appeared to be high at baseline. There was little change in overall BF and CF knowledge among community volunteers. Similar to training exposure, knowledge scores were consistently lower in SNNPR than in Tigray. In general, supervisors had the highest mean BF knowledge scores, followed by HEWs and volunteers. Mean CF knowledge scores were similar among supervisors and HEWs, and higher than those for volunteers. When specific knowledge items were compared, several patterns emerged. BF knowledge was high even at baseline, but knowledge about BF frequency (“breastfeeding the baby on demand/cue”) increased markedly among supervisors and HEWs, as well as knowledge about breast milk sufficiency (“breastfeeding more often if mother thinks baby is not getting enough”) among supervisors. Among all FLWs, CF knowledge about adding egg or a special food to the baby’s porridge increased substantially; this was a key message promoted by the A&T program.

**Table 4:** **IYCF knowledge among frontline workers by region and survey year**

	**2010**		**2013**	
**Indicators**	**Percent**		**Percent**	
	**Tigray**	**SNNPR**	**Tigray**	**SNNPR**
**Supervisor knows:**	**(n = 25)**	**(n = 48)**	**(n = 25)**	**(n = 46)**
Putting the baby on breast immediately or <1 h after birth	92.0	89.4	96.0	97.9
Giving colostrum to the baby	92.0	97.9	100.0	95.7
Not giving water, even in hot weather	96.0	89.4	100.0	95.7
Breastfeeding the baby on demand/cue	56.0	63.8	92.0	76.1
Breastfeeding more often if mother thinks baby is not getting enough milk	56.0	48.9	84.0	63.0
BF knowledge score range (5 total items)	2-5	1-5	3-5	2-5
**BF knowledge score (mean, SD)**	**3.9 (0.8)**	**3.9 (0.9)**	**4.7 (0.5)**	**4.3 (0.9)**
Introducing complementary foods at 6 mo	100.0	95.7	92.0	95.7
Problem of gruel that is too thin	68.0	55.3	96.0	76.1
Adding egg or special foods to baby’s porridge	28.0	12.8	72.0	37.0
No. of times children aged 12–23 mo need complementary foods	92.9	100.0	100.0	94.7
CF knowledge score range (4 total items)	1-4	1-3	1-4	1-4
**CF knowledge score (mean, SD)**	**2.4 (0.7)**	**2.0 (0.7)**	**3.4 (0.8)**	**2.5 (0.8)**
	**Tigray**	**SNNPR**	**Tigray**	**SNNPR**
**HEW knows:**	**(n = 25)**	**(n = 48)**	**(n = 40)**	**(n = 93)**
Putting the baby on breast immediately or <1 h after birth	84.0	91.7	100	98.9
Giving colostrum to the baby	88.0	89.6	87.5	78.5
Not giving water, even in hot weather	92.0	91.5	97.5	97.8
Breastfeeding the baby on demand/cue	64.0	58.3	72.5	74.2
Breastfeeding more often if mother thinks baby is not getting enough milk	52.0	41.7	55.0	55.9
BF knowledge score range (5 total items)	1-5	2-5	2-5	2-5
**BF knowledge score (mean, SD)**	**3.8 (0.9)**	**3.7 (0.9)**	**4.0 (0.9)**	**4.0 (0.8)**
Introducing complementary foods at 6 mo	96.0	97.9	95	93.6
Problem of gruel that is too thin	92.0	66.7	77.5	83.9
Adding egg or special foods to baby’s porridge	20.0	14.9	57.5	50.5
No. of times children aged 12–23 mo need complementary foods	100.0	84.6	100.0	97.0
CF knowledge score range (4 total items)	1-4	1-3	1-4	1-4
**CF knowledge score (mean, SD)**	**2.7 (0.7)**	**2.0 (0.7)**	**3.1 (0.9)**	**3.0 (0.8)**
	**Tigray**	**SNNPR**	**Tigray**	**SNNPR**
**Volunteer knows:**	**(n = 25)**	**(n = 48)**	**(n = 104)**	**(n = 196)**
Putting the baby on breast immediately or <1 h after birth	92.3	100.0	88.5	93.4
Giving colostrum to the baby	88.5	87.8	85.6	78.6
Not giving water, even in hot weather	69.2	71.4	82.5	81.3
Breastfeeding the baby on demand/cue	65.4	56.3	56.7	57.7
Breastfeeding more often if mother thinks baby is not getting enough milk	19.2	34.7	32.7	35.7
BF knowledge score range (5 total items)	0-5	0-5	0-5	1-5
**BF knowledge score (mean, SD)**	**3.3 (1.2)**	**3.3 (1.2)**	**3.5 (1.1)**	**3.4 (1.1)**
Introducing complementary foods at 6 mo	76.0	72.9	97.1	98.5
Problem of gruel that is too thin	76.0	64.6	70.2	62.8
Adding egg or special foods to baby’s porridge	12.0	4.1	42.3	43.9
No. of times children aged 12–23 mo need complementary foods	93.3	100.0	95.9	99.1
CF knowledge score range (4 total items)	0-3	0-4	1-4	1-4
**CF knowledge score (mean, SD)**	**2.1 (0.7)**	**1.7 (0.9)**	**2.8 (0.9)**	**2.6 (0.9)**

There were very few negative self-reported perceptions about work or job performance (data not shown). In both regions, 76.5% to 96.0% of all FLWs agreed or strongly agreed on their confidence about job performance, serving as a reference for overall work-related self-efficacy. FLWs in Tigray (72.1% to 88.0%) expressed more confidence about their ability to provide IYCF education, compared to their counterparts in SNNPR (59.2% to 67.8%). Community volunteers were the least confident about their ability to give IYCF education in either region. Most FLWs also reported the need for further training on IYCF counseling, with the highest response rate (92.3%) among volunteers in SNNPR. These results corroborated the trends in IYCF knowledge among different FLW types and between regions.

Training participants responded positively about the A&T training content and delivery. Three particular aspects were highlighted by HEWs: (1) the succinct messages of the 7 Excellent Feeding Actions (“*I really liked it because the 7 feeding activities are compiled together.*”); (2) practical activities such as the food preparation demonstrations (“*What I liked most is the preparation of the soft thick porridge. It really makes a difference when they show in practice.*”); and (3) the focus on fathers’ involvement in child feeding. Community volunteers also reinforced these aspects, particularly the usefulness of practical food demonstrations and the focus on locally available foods. While no respondent directly expressed negative opinions about the training, 3 of the 12 HEWs and 7 of the 36 volunteers interviewed reported the need for more frequent or continual training to help them retain the information received.

### Dissemination of program tools and messages (Figure 1, component b)

#### Adherence and exposure to program tools

Similar to training, program tools are intended to be disseminated in a cascading manner. Copies of program tools are distributed during training to the supervisors and HEWs, who provide counseling and disseminate the materials to mothers and other caregivers directly and via the support of community volunteers. The primary tool used for counseling and dissemination to households is called Tool B or child nutrition card, a poster containing a brief message and image for each of the 7 Excellent Feeding Actions. This IPC tool and the 7 Actions were used as “tracers” in the delivery of program tools and messages.

**Figure 2:**
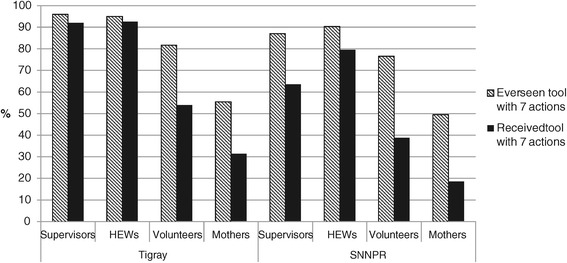
**Exposure to IPC tool by respondent type and region, 2013.**

In Tigray, most supervisors and HEWs had received copies of the IPC tool (92.0% and 92.5% respectively), but only 53.9% of volunteers had received the tool despite 81.7% having seen it (Figure 2). The pattern of exposure was similar in SNNPR but at lower percentages. Nearly all of the supervisors and HEWs received the tool during training. Among the volunteers who had the tool, most reported receiving it from HEWs. Few HEWs reported that a shortage of materials was the reason for not disseminating. Among volunteers who did not receive the tool, several understood that it was for use exclusively by HEWs: “*I don’t have it myself, but I have seen it. I saw B card [Tool B], but I don’t know the importance… They [HEWs] didn’t give it to us; they distribute.*” “*I don’t know what the reason is. Maybe it is because I didn’t go to the health post recently that they didn’t give it to me. HEWs distribute this tool to mothers in my village when they go for vaccination, but they didn’t give it to me to teach mothers. I teach mothers only based on what I heard from my previous training about child feeding and care.*” Thus, there was a gap in the communication between HEWs and volunteers about the purpose and use of the program tools.

In turn, exposure to the IPC tool was low among mothers, despite increases in the overall number of contacts with HEWs and volunteers in both regions since baseline (data not shown). Only 55.4% of mothers in Tigray and 49.5% in SNNPR had ever seen the tool, with far fewer (31.4% in Tigray and 18.5% in SNNPR) having received a copy to keep as a reminder in their homes (Figure 2). Among mothers who had seen the tool, the main source was HEWs; only 4.2% of mothers in Tigray and 3.4% in SNNPR had seen the tool from community volunteers.

#### Quality and responsiveness to program tools

Most HEWs and volunteers who had the IPC tool reported using it regularly (once to at least three times a week) to provide education and counseling to beneficiaries (data not shown). The main reasons for not using the IPC tool more often were related to the issue of time; FLWs perceived that either they or the mothers had insufficient time to use the tool to discuss its content.

**Figure 3:**
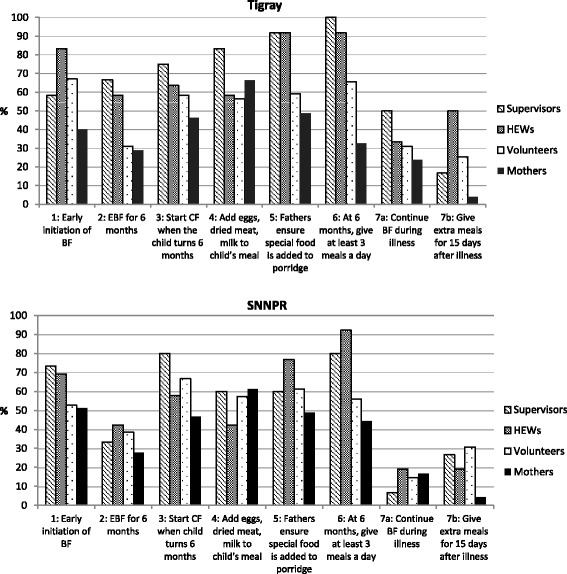
**Aided recall of the 7 key program messages by respondent type and region, 2013.**

Delivery quality was also assessed through open recall of the 7 Excellent Feeding Actions and knowledge of the messages using recall aids (pictures of the 7 Actions) among those who were exposed to the tool. Figure 3 presents the proportions of correct responses based on aided recall. In Tigray, more than 50% of supervisors and HEWs correctly identified six out of the seven key messages. Knowledge of the messages was lower among community volunteers. Trends were similar in SNNPR but at lower percentages. Despite positive opinions about the usefulness and aesthetics of the tool among FLWs (“*This tool is prepared in an easily understandable way. Previous tools used written messages only, while this one has attractive pictures and written messages. Even illiterate mothers can easily understand it very well by looking at the pictures and practice.*”), they showed partial knowledge of the tool’s contents. The patterns of message recall among mothers were similar to but more attenuated than those among FLWs.

### Supervision and feedback (Figure 1, component c)

#### Supervision adherence and exposure

The feedback loop in service delivery flow is the supportive supervision provided to FLWs. This component is particularly important since HEWs are responsible for delivering 17 different packages of health services (among which nutrition is one), thus requiring guidance and motivation to execute all activities as intended. On average, each HEW supervisor supervises 6–8 HEWs, and each pair of HEWs in turn supervises 50 community volunteers in their kebele. These personnel ratios were pre-established by the government and not defined by the program. HEW supervisors schedule monthly visits to health facilities and visit selected households in order to assess and reinforce service delivery (e.g. observing counseling sessions, orienting about IYCF, checking stocks of IPC tools and supplies, etc.) and provide immediate feedback. Nearly all of the supervisors in Tigray reported providing supervisory visits with observations of BF and CF counseling at health posts and group counseling sessions in the past year, an increase from 80% at baseline (data not shown). In SNNPR, 76.1% of supervisors reported observing BF counseling and 78.3% observed CF counseling at health posts and group sessions, which were similar to baseline.

However, only 52.5% of HEWs in Tigray and 33.7% in SNNPR reported having received supervisory visits in the past one month, and some reported never receiving any supervisory visits (5.0% in Tigray and 10.8% in SNNPR). Among community volunteers, 41.8% in Tigray and 21.8% in SNNPR received supervision in the past month. Volunteers who received supervision reported receiving visits primarily from HEWs, but often irregularly at a few times a year or at unspecified frequency.

### Supervision quality and responsiveness

Among HEWs who received supervision, most confirmed that visits included orientation about IYCF information (92.1% in Tigray and 90.5% in SNNPR). However, activities such as checking the availability of IPC tools (31.6% of HEWs in Tigray and 20.3% in SNNPR) or providing tools (23.7% and 6.8% respectively) and providing immediate feedback (7.9% and 6.8% respectively) were reported less often. Similar patterns of activities covered in supervision were reported by community volunteers. Supervision received by volunteers were conducted individually or in groups and often included orientation about IYCF information (95.7% in Tigray and 93.0% in SNNPR), but little else of other activities related to IYCF materials or corrective actions. Qualitative findings reinforced that supportive supervision did not cover all the expected elements. Supervisees were routinely checked for completed activities and given some technical information, but the availability and use of materials were not checked, and they rarely received advice on ways to improve on their mistakes or how to complete their activities under constraints.

## Discussion

Our study examines evidence of different degrees of implementation fidelity along the delivery pathways of A&T’s community-based IYCF intervention program. As a major programmatic component, FLW training on IYCF was carried out with concentrated effort and resources, as reflected in its strong adherence to the cascading schema of the program design with high exposure at every FLW level and positive trends in quality and participant responsiveness. However, FLWs also received nutrition training from other sources such as the government-supported CBN program. Thus without specific training observations or evaluations or comparable measurements from a control group, our findings of increased IYCF knowledge and work-related self-efficacy may not be attributed to the A&T program training, despite it likely having made a contribution. The partial adherence in the dissemination of program tools and messages, particularly to the volunteers and mothers, highlights the gap between the formal health system and community levels. Community volunteers appeared to remain underutilized in the program delivery chain. Despite the highly positive responses about the appearance and usefulness of the program tools, these opinions appeared to have little effect on the adherence to their proper use and delivery. FLWs who were exposed to the program tools had correct knowledge of several key program messages, but poor aided recall of certain messages and the lower levels of knowledge among volunteers and mothers further signal weak delivery quality. Supportive supervision was intended in tandem with training and dissemination of program tools and messages to address these gaps in delivery and capacity among FLWs. However, there was low supervision fidelity across the FLW levels. Despite a feasible ratio of supervisor to HEWs in place, monthly supervision among HEWs was low; supervision of volunteers by HEWs faced a greater challenge, given the large numbers of volunteers under the responsibility of few HEWs.

In addition, there were regional differences for nearly all measures. Given little difference in the participant characteristics between regions, the main variations are likely in program intensity. In Tigray the primary A&T partners, IFHP and REST, have strong technical capacities in the areas of health and nutrition. On the other hand, in SNNPR, the most rural region of Ethiopia, women’s association and the evangelical church were the primary partners and had more limited levels of technical capacity. These factors likely play an important role in the ability to reach beneficiaries and provide quality service delivery.

FLWs’ work context may be a factor influencing the apparent program delivery gap between the health system and communities. Although not presented here, time and workload were identified in our study as work constraints particularly among HEWs. These are not surprising findings, as they align with results from other studies on the challenging work conditions of HEWs and implementation of the Health Extension Program [10,25]. Under these conditions, delivering IYCF education that require sustained and frequent outreach to families and skills in behavior change communication (BCC) may face even greater difficulties. Without substantial adjustments in work structures, it would be difficult for HEWs to provide services that are demanding in terms of time and complex skills with much success or to train and supervise volunteers to help share and ease their workload. Although the new cadre of community volunteers is intended to achieve better reach of information and services in the communities, their turnover may have also affected program adherence and exposure.

Carroll et al.’s [15] framework for implementation fidelity considers a fifth aspect of *program differentiation*, or identifying the unique and essential features of programs. We did not explicitly draw out this element, as we considered the “essential components” to be embedded in our study as the program tracers, i.e. the 7 Excellent Feeding Actions. Given the extensive and complex information base for IYCF, A&T conducted formative work to distill them into these 7 Actions, which were the key messages delivered primarily through the FLWs and discussed in our results of dissemination of program tools and messages. However, there are differential uptake and utilization even among these seven actions, which will be addressed later in relation to the effect on impact outcomes.

There are some limitations to this study. First, the retrospective measurements of adherence and delivery quality are limited by recall bias. Our primary measure of adherence was exposure to intended source and by proportions at each participant level. Although content was traced to verify specific program components, it was not measured through direct observations or evaluations. Frequency and duration were obtained wherever possible, but they were not systematically measured for the different components due to measurement difficulties, poor recall, and inconsistent reporting. However, we applied multiple methods to study the different elements of fidelity, in order to corroborate results as often as possible. Second, there was no comparison group in our study, against which to compare measures of training quality, knowledge, supportive supervision, etc. This limits attributions to the program, and we acknowledge the effects of this in the interpretation of our results. Third, the duration of full implementation and exposure to different program components was shorter than expected, at approximately one year, and not uniform. As previously discussed, the program realized a shorter implementation period than intended due to national policy changes in the training process and structure of community volunteers. Shorter exposure in some areas may result in underestimation of effects. For this reason, we assess only the delivery side of the program impact pathways in this study, from provision of inputs to knowledge, where most changes should be detected. We do not assess for any changes further down the impact pathways such as practices and nutritional status among beneficiaries, which will be evaluated later in the 2014 endline survey. Lastly, the summary scores for IYCF knowledge used as measures of training quality are not standardized, so it is difficult to interpret the meaning of differences and to know what magnitudes of difference affect outcomes such as skills and practice. However, rather than interpreting their effects, we used their relative results to make comparisons over time (2010 baseline to 2013 PE surveys) and across different FLW levels. Determining the meaningful size of differences in knowledge scores and other process-related quality indicators is an area for further research [26].

Our findings suggest that while fidelity in program training has been high, gaps remain in the delivery of program tools and messages to beneficiaries, directly by HEWs and via community volunteers, as well as in the supervision of HEWs and volunteers. Unless these gaps are addressed, it is unlikely that the expected impact at household level will be observed at scale. Given that the delivery of the BCC intervention for improved IYCF requires a sequence of programmatic components and actions to work together, the use of program impact pathways helped to identify how and where the components are or are not implemented as intended. Our study adds to the growing literature on theory-driven process evaluations that assess factors influencing the effectiveness of different nutrition interventions [17,27-29]. Also, applying mixed methods to assess different domains of fidelity allowed us to corroborate and help interpret findings of different measures, thereby strengthening the internal validity of our results.

Our study findings were reported back to the program to address issues related to the ongoing program. However, as the program was preparing to phase out in its current areas by 2014, these findings were considered in developing the design for a second phase of the program to be initiated in 2015. Furthermore, given the longer duration and extensive fieldwork of the process evaluation, this study was not intended to replace routine monitoring for timely feedback and corrective actions. Still, the process evaluation helps to understand critical elements of the implementation process and the conditions and factors influencing the process, and sheds light on how the program might eventually achieve its intended impact.

## Conclusions

In Tigray and SNNPR, the volunteer cadre is underutilized and presents the greatest challenge to reaching mothers of infants and young children. To maximize the scale and reach of the health extension platform to improve IYCF, efforts should focus on ensuring that the volunteer corps is trained, adequately supported and supervised by the HEW, and in possession of the required materials for delivering BCC to the households in their catchment area. To do so may require a combination of approaches, for example: 1) more intense supervision of the HEWs and volunteers, particularly with explicit instructions and better guidance on supervision and a more feasible ratio of HEWs to volunteers, potentially through intermediary roles by leaders over teams of volunteers; 2) performance-based incentives to motivate these cadres of health workers; 3) a reassessment of the workloads of these cadres and task shifting to ensure that delivery of the expected IYCF interventions is manageable for both; and 4) simplifying the IYCF interventions, for example, by targeting fewer IYCF behaviors (or at least prioritizing those age-specific practices that require greater improvement). Only by strengthening the linkages between the HEW and the volunteers is it likely that this platform will reach the target households and deliver IYCF results at scale.

## Endnotes

^a^WDAs, recently referred to as “1for5s,” are women of reproductive age within a village, with one leader responsible for checking and following up on the status of hygiene, childcare practices, health, nutrition, and other topics among a group of five other women. WDAs exclusively working on health topics are also called HDAs, particularly in Tigray.

^b^The maternal and child health module was one of five modules to be rolled out under IRT in 2012. Training on different modules are conducted on a rotation basis, and IRT on the maternal and child health module was conducted once per region and not repeated by the time of this study.

## Abbreviations

A&T: Alive & Thrive
BF: Breastfeeding
CBN: Community-Based Nutrition
CF: Complementary feeding
ENA-BCC-CF: Essential Nutrition Actions-Behavior Change Communication-Complementary Feeding
FLW: Frontline worker
HDA: Health Development Army
HEW: Health extension worker
IFHP: Integrated Family Health Program
IPC: Interpersonal communication
IYCF: Infant and young child feeding
IRT: Integrated Refresher Training
PE: Process evaluation
REST: Relief Society of Tigray
SNNPR: Southern Nations, Nationalities and Peoples Region
WDA: Women’s Development Army
